# A Review of Metabolic Targets of Anticancer Nutrients and Nutraceuticals in Pre-Clinical Models of Triple-Negative Breast Cancer

**DOI:** 10.3390/nu14101990

**Published:** 2022-05-10

**Authors:** Alleigh Wiggs, Sabrina Molina, Susan J. Sumner, Blake R. Rushing

**Affiliations:** 1 Department of Nutrition, University of North Carolina-Chapel Hill, Durham, NC 27599, USA; 2 Nutrition Research Institute, University of North Carolina-Chapel Hill, Kannapolis, NC 280821, USA

**Keywords:** breast cancer, treatment, metabolism, metabolic reprogramming, nutrients, metabolomics, nutraceuticals

## Abstract

Triple-negative breast cancer (TNBC) is a subtype of breast cancer that is notoriously aggressive and has poorer outcomes as compared with other breast cancer subtypes. Due to a lack of targeted therapies, TNBC is often treated with chemotherapeutics as opposed to hormone therapy or other targeted therapies available to individuals with estrogen receptor positive (ER+) breast cancers. Because of the lack of treatment options for TNBC, other therapeutic avenues are being explored. Metabolic reprogramming, a hallmark of cancer, provides potential opportunities to target cancer cells more specifically, increasing efficacy and reducing side effects. Nutrients serve a significant role in metabolic processes involved in DNA transcription, protein folding, and function as co-factors in enzyme activity, and may provide novel strategies to target cancer cell metabolism in TNBC. This article reviews studies that have investigated how nutrients/nutraceuticals target metabolic processes in TNBC cells alone or in combination with existing drugs to exert anticancer effects. These agents have been shown to cause perturbations in many metabolic processes related to glucose metabolism, fatty acid metabolism, as well as autophagy and oxidative stress-related metabolism. With this information, we present the potential of nutrients as metabolism-directed anticancer agents and the potential for using these agents alone or in cocktails as a new direction for TNBC therapy.

## 1. Introduction

Triple-negative breast cancer (TNBC) characterizes breast cancers with a lack of estrogen/progesterone receptors and human epidermal growth factor receptor 2 (HER2), making it more difficult to treat than estrogen receptor positive (ER+) breast cancers [1]. In the US, TNBCs makes up about 10–15% of all breast cancers diagnosed, and are most common in women under 40 years of age, women with a BRCA1 mutation, and rates are disproportionately higher in African American women [2]. The 5-year survival rate decreases as cancer moves outside of the breast, from a 91% 5-year survival rate when the cancer tissue is localized, to a 12% 5-year survival rate when the cancer has reached other tissues [2]. This may be compared with ER+ breast cancers, which overall have better survival rates, in part due to increased treatment options. Additionally, TNBC is typically discovered in later stages or after it has metastasized, making treatment more difficult and less effective [1,2].

Current guidelines for treating stage I–III TNBC include lumpectomy surgery or mastectomy, and radiation therapy. This may be paired with adjuvant chemotherapy drugs which may include classes of anthracyclines, taxanes, cyclophosphamides, and carboplatin drugs [3]. In more advanced stage cancers, chemotherapy may be administered before surgery to improve surgical outcomes. In addition, certain TNBC patients may benefit from immunotherapy approaches [4]. Compared with many other cancers, few new treatment options for TNBC have emerged over the last several decades [5]. Therefore, there is a need to explore new therapeutic strategies to replace or augment existing treatments to improve TNBC outcomes and/or reduce side effects.

In 1920, Otto Warburg observed that cancer cells took in larger amounts of glucose than typical somatic cells and used more anaerobic glycolysis as a means of energy production, a phenomenon now known as the “Warburg Effect” [6]. Other phenotypes of malignant cells would later be discovered and may provide more precise therapeutic targets for cancer therapy. Most recently research has focused on using metabolic-directed therapy to slow or arrest cancer growth. For example, methotrexate, which may be used for cancer or rheumatoid arthritis treatment, works to competitively inhibit dihydrofolate reductase, limiting the amount of tetrahydrofolate present in the cell [7]. The success of drugs such as methotrexate supports the idea that targeting cell metabolism may be a viable way to treat cancer. Additionally, metabolic reprogramming is a common characteristic of cancer cells [8]. Indeed, it is now well-recognized that cancer cells modify cellular metabolism to support increased anabolic processes, improve cancer cell survival in tumor environments such as hypoxia, and facilitate many steps in cancer progression such as angiogenesis, intravasation/extravasation, and immune evasion [9,10,11,12,13,14].

The aggressive nature of TNBC and lack of targeted therapies has highlighted the need for further investigation into therapies that work by targeting cellular metabolism, including nutraceutical therapy. Nutrition plays an influential role in the metabolism of a cell because essential amino acids, lipids, carbohydrates, minerals, and vitamins/co-factors are involved in the transcription and regulation of genes, DNA methylation, protein folding and protein modification, regulation of microbial/gut metabolism, and hundreds of biochemical reactions of host metabolism. However, not enough is known about how nutrients can be used to modulate the metabolism of TNBC to facilitate cancer cell death and improve patient outcomes. This review will discuss major metabolic phenotypes of TNBC, and how certain nutrients have been shown to experimentally target these pathways to exert anticancer effects in TNBC cells.

## 2. Methods

Our goal for this review article was to identify nutrients/nutraceuticals that are known to target TNBC metabolism to exert an anticancer effect. To do this, the literature was first surveyed for metabolic programming phenotypes characteristic of TNBC. No restrictions were placed on publication date. Based on the TNBC metabolic features that were observed in these previous studies, a literature search was performed through PubMed to identify nutrients/nutraceuticals that targeted these metabolic pathways in TNBC models. Searches were performed with the following key phrases: “diet breast cancer glycolysis” “nutrient breast cancer glycolysis” “diet triple-negative breast cancer treatment” “diet breast cancer pyruvate” “nutrition breast cancer pyruvate” “nutrition breast cancer glutamate” “diet breast cancer glutamate” “nutrition breast cancer fatty acid oxidation” “diet breast cancer fatty acid oxidation” “breast cancer fatty acid oxidation supplement” “breast cancer glycolysis supplement” “breast cancer pyruvate supplement” “triple-negative breast cancer nutrition supplements” “triple-negative breast cancer lipid metabolism treatment nutrient” “anti-cancer nutrition supplement” “anticancer nutrition glycolysis” “anticancer nutrition lipid metabolism” “anticancer nutrition metabolism triple-negative” “dietary breast cancer triple-negative.”

This search yielded 915 studies which were imported into COVIDence and filtered based on our inclusion and exclusion criteria. Inclusion criteria included studies focused on established TNBC models, whether in vivo or in vitro, that had clear measurements of metabolites following nutrient/dietary compound treatment. Studies that only observed anticancer effects (i.e., cell death, reduced rate of proliferation) without looking at metabolic pathway effects were excluded. Initially, titles and abstracts were screened to identify studies that passed the inclusion/exclusion criteria and to remove duplicate results. Full-text reviews were then performed to assess overall study quality as well as the quality of methods to measure metabolites/metabolic pathways. Information extraction was performed manually for studies that passed this process for incorporation into our review of metabolism-targeting compounds.

## 3. Results

### 3.1. Glycolysis

The Warburg effect is often a hallmark of many cancer cells, and this metabolic phenotype is also seen in triple-negative breast cancer [7,8]. While oxidative phosphorylation may have a larger energy yield, lactic acid fermentation may be a faster process, allowing for more rapid energy production for proliferating cells [8]. Additionally, the accumulation of pyruvate and upstream metabolites also provides substrates for other cellular pathways, including the pentose phosphate pathway, for anabolic reactions [10].

Metabolic reprogramming seen in TNBC helps to facilitate this Warburg phenotype. Increased glucose uptake via GLUT-1 transporters is a key characteristic of TNBC [15]. USP6NL levels are overexpressed in many breast cancer types, including triple-negative, which in turn leads to chronic AKT activation through phosphorylation [16]. AKT has been found to stabilize GLUT-1 transporters—the primary glucose transporter in mammary cells—in the plasma membrane, allowing for more access to glucose for metabolism [16].

In TNBC specifically, Epidermal Growth Factor (EGF) signaling is typically dysregulated and TNBC patients commonly express the transmembrane EGF receptor [17]. EGF signaling has been found to activate the first step in glycolysis, and downstream metabolites may also increase this effect [18]. This increase in signaling may also induce the phosphoinositide 3-kinase (PI3K)/AKT pathway, which in turn leads to increased hypoxia-inducible factor 1 alpha (HIF-1a) levels [19]. HIF-1a leads to many downstream effects, including changing the fate of intracellular glucose, which leads to increased rates of anaerobic glycolysis [20,21]. Thus, research into compounds that augment glucose uptake and utilization or alter aerobic glycolysis signaling are of particular interest.

#### 3.1.1. PI3K and AKT

A 2019 study by Roy et al. investigated the effects of benzyl isothiocyanate (BITC) on TNBC cells, as preliminary research showed inverse relationships between increased cruciferous vegetable consumption and cancer. BITC administration led to increased GLUT-1 localization to the plasma membrane, and increased activation of AKT through Akt phosphorylation in cell cultures and mice models [22]. BITC administration thus induced glucose dependency, which creates a possible druggable target. Roy et al. found that with AKT pharmacological inhibition, there was an increased rate of apoptosis in the breast cancer cells [22].

A 2015 study by Shrivastava et al. showed that celasterol, present in the root extracts of Tripterygium wilfordii and Tripterygium regelii, decreased phosphorylation of AKT, even in the presence of insulin [23]. Celasterol was found to reduce signaling of the PI3K/ATK/mTOR axis, and was found to induce apoptosis, possibly through this mechanism. While not addressed by this study, further research could be done on the effects of glucose uptake as celasterol was shown to decrease ATK phosphorylation, which may in turn decrease GLUT-1 stabilization in the plasma membrane [23].

#### 3.1.2. HIF-1a

Cardamonin, a chalcone found in Alpiniae katsumadai, has been shown to downregulate HIF-1a mediated cell activity [24]. HIF-1a mRNA and protein levels were both decreased in treated cells, attributed to cardamonin’s ability to repress the mTOR/p70S6K pathway. Additionally, the study found an increase in reactive oxygen species (ROS) and increased apoptosis. Other glycolytic markers such as glucose uptake and lactic acid production were also decreased [24].

Mouradian et al. showed that docosahexaenoic acid (DHA) supplementation in vitro led to a dose-dependent decrease in HIF-1a protein levels in MDA-MB-231 cells (a commonly used human epithelial TNBC cell line) [25]. Additionally, lactate dehydrogenase was downregulated, and total lactate production was decreased in MDA-MB-231 cells treated with DHA. In this study, GLUT-1 protein was not regulated (possibly due to the high levels of expression regardless of HIF-1a), although total glucose uptake was decreased in cells treated with DHA. However, these effects were not seen in a non-transformed cell line, MCF-10a. Thus, DHA may contribute to reversing the Warburg phenotype present in TNBC cells, while leaving nonmalignant cells unaffected [25].

A similar cascade was seen in a study by Santos et al., where MDA-MB-231 cells were treated with Vitamin D3 (calcitriol) [26]. At 0.5 umol, Vitamin D3, GLUT-1 and lactate dehydrogenase A (LDHA) expression were decreased. At 1 umol, Vitamin D3, Hexokinase II and LDHA expression decreased. The study also found that administration of both doses, glucose uptake was decreased, and at the 1 umol dose, lactate production was also decreased. Lastly, administration of Vitamin D3 to the highly metastatic MDA-MB-231 cells led to decreased cell proliferation and increased apoptosis compared with the controls [26].

### 3.2. Fatty Acid Metabolism

TNBC is known to have greater rates of fatty acid uptake, de novo synthesis, and fatty acid oxidation to support proliferation [8,27]. TNBC cells increase their fatty acid stores through breakdown of circulating triglycerides, or from de novo synthesis [8]. Lipoprotein Lipase has been found to be highly expressed in TNBC cells [28], allowing dietary fatty acids to contribute to lipid stores. Fatty acid synthesis is another key contributor to the lipid supply of malignant cells, with many cancers exhibiting increased expression of many lipogenic enzymes, including fatty acid synthase (FAS) [29].

#### 3.2.1. Fatty Acid Synthase

In a 2016 study, Xiao et al. found that leucine suppressed fatty acid synthase and the sterol response element protein C1 expression in TNBC cell lines and xenograft mice, as measured by mRNA concentration [30]. These treated cells also had decreased concentrations of palmitate, the key product of FAS which is encoded by the fatty acid synthase (*FASN)* gene. However, when supplemented with palmitate over four days, further tumor growth was seen as compared with mice supplemented with a vehicle control. Additionally, although administration of a leucine-free diet also led to decreased cell viability in the TNBC cell culture, non-malignant cell types were unaffected [30].

(-)-Epigallocatechin 3-gallate (EGCG) and its derivatives are compounds found to regulate fatty acid synthesis [31]. Crous-Maso et al. used EGCG analogues in the form of diesters and monoesters to investigate cytotoxicity of the compounds on TNBC cell lines. They found that treatment of cells with the analogues led to decreased FAS activity, which was measured by expression of the FAS gene (*FASN)*. Similarly, the G28 diester and M1 and M2 monoesters were found to decrease overall fatty acid synthase protein levels. Administration of the monoesters also led to apoptosis. Again, introducing palmitate led to increased cell viability [31].

#### 3.2.2. Stearoyl CoA Desaturase and Lipid Droplets

Stearoyl CoA desaturase is another important enzyme involved in lipid metabolism in cancer cells. Stearoyl CoA desaturase catalyzes the formation of unsaturated fatty acids [32], which in turn provides a supply of phospholipids which may be incorporated into the proliferating cell’s plasma membrane. Increased expression is also associated with poorer outcomes for breast cancer patients [33].

In a 2018 study, anacardic acid was found to inhibit proliferation of MDA-MB-231 cells. The transcriptomic response was then evaluated. Anacardic acid was found to decrease stearoyl CoA desaturase transcription amongst other proteins and is thought to serve a role in limiting monounsaturated fatty acid synthesis in TNBC cells [34].

Lipid droplets (LD) serve as protective cell components which increase TNBC cell survival in times of starvation [35]. In parallel, increased lipid droplet accumulation is associated with more aggressive breast cancer types [36]. Targeting lipid droplet synthesis and degradation may restrict the malignant tissue’s ability to proliferate, and possibly lead to a less aggressive phenotype.

Pizato et al. measured the effect of DHA and vitamin E delta-tocotrienol (Delta-T3) on lipid droplet biogenesis and breakdown. DHA and Delta-T3 co-treatment was found to decrease lipid droplet biogenesis and increase lipid droplet lipophagy in MDA-MB-231 cells [37]. However, when administered without Delta-T3 co-treatment, DHA was found to stimulate lipid droplet biogenesis [37].

### 3.3. Autophagy, Apoptosis, and Oxidative Stress

Dysregulation of autophagy and apoptosis signals can lead to breast cancer cell initiation and survival [8]. In the absence of cancer, autophagy is used to recycle damaged proteins and organelles through formation of the autophagosome and subsequent degradation [38]. However, with the onset of cancer initiation, autophagy is used to provide substrate for the proliferating cell. Certain cellular pathways engage in signaling autophagy, including the mTOR pathway, which is also involved in other metabolic processes previously highlighted [39]. The following studies investigated the role of nutraceutical supplementation in increasing oxidative stress and inducing apoptosis.

Vibet et al. in 2011, reported that DHA and anthracycline treated MDA-MB-231 cells had increased levels of reactive oxygen species (ROS), decreased glutathione peroxidase activity, and accumulation of glutathione [40]. The study concluded that DHA sensitizes the TNBC cells to anthracyclines, making treatment more effective. These effects were reversed with the administration of Vitamin E [40].

In a 2015 study by Tran et al., a blend of tocotrienols and tocopherols (marketed as Tocomin) was administered to MDA-MB-231 and MCF-7 cells. The study found decreased rates of cell proliferation, increased rates of apoptosis, and negative modulation of the PI3K and mTOR pathways by Tocomin [41]. As part of their investigation, the researchers investigated if Tocomin-induced autophagy was part of the apoptotic process. Tocomin was found to induce autophagy in MDA-MB-231 cells and administration of an autophagy inhibitor 3-methyladenine (3-MA) potentiated the cytotoxic effect of Tocomin, suggesting that autophagy played a protective role in breast cancer cells in response to Tocomin treatment [41].

St. John’s Wort was also found to induce autophagy and apoptosis in MDA-MB-231 cells [42]. Anti-apoptotic proteins were downregulated and the p-PI3K/PI3K and p-mTOR/mTOR ratios were also suppressed. When autophagy was suppressed with 3-MA, cell proliferation increased and rates of apoptosis reversed, indicating that St. John’s Wort may work on the pro-death autophagy pathway. When studied in MDA-MG-231 xenografted athymic nude mice, administration of St. John’s Wort induced autophagy and apoptosis, as well as in vivo tumor growth inhibition [42].

In 2020, Lin et al. investigated the effects of isoliquiritigenin (ISL, a compound found in licorice) on cellular apoptosis. The study found that with the administration of ISL, cell cycle progression was halted via decreased cyclin D1 expression [43]. Markers of apoptosis including decreased Bcl-2 protein and increased Bax protein expression were also seen in the experimental group. Additionally, total and phosphorylated levels of mTOR were also downregulated. In mouse models, treatment with ISL also led to decreased tumor weight compared with controls [43].

Antrodia Salmonea (AS), a Taiwanese mushroom and nutraceutical, has been shown to induce cell cycle arrest. In 2017, Chang et al. found that administration of AS to MDA-MB-231 cells led to increased G2 cell cycle arrest [44]. They also found decreased numbers of G1 and S phase cells, indicating that AS may induce apoptosis alongside cell cycle arrest. Cyclin B1, cyclin A, cyclin E, and CDC2 were all downregulated in the treatment groups, indicating that AS may act on the G2 transition. COX-2 protein expression decreased, and PARP cleavage was also induced with AS supplementation. The co-administration of N-acetylcysteine, an ROS inhibitor, with AS prevented reductions in cyclin B1, cyclin A, cyclin E, CDC2, and COX-2 protein expression than were measured with AS treatment alone. In xenografted nude mice, administration of AS also led to decreased tumor progression. The in vivo tumor models also showed an accumulation of LC3B-II and an increase in caspase-3 [44]. In a second 2017 publication, Chang et al. reported again that AS led to increased TNBC apoptosis and autophagy [45]. With administration of AS, LC3-II levels and AVOs formation increased. Associated with this, levels of ATG7 increased, p-mTOR decreased, SQSTM1/p62 expression was reduced, and Beclin-1/Bcl-2 ratio regulation was disrupted. Co-treatment with compounds that diminished ROS accumulation, autophagy, or apoptosis also reversed the effects seen with administration of AS [45].

The effects of the phloretin, a dihydrochalcone found in apples was invested by Chen et al., 2021, using MDA-MB-231 breast cancer cells [46]. In glucose-limited media, phloretin led to decreased cell growth. Similarly, in the glucose-restricted media, phloretin inhibited formation of the autophagosome and inhibited LC3B-1 to LC3B-II conversion. The study also found a decrease in ULK1 protein expression in affected cells, as well as an increase in phosphorylated forms of mTOR and AMPK. Co-treatment with chloroquine and 3-MA also led to a further decrease in cell viability. Lastly, the study found an increased sensitivity to doxorubicin in nude mice transfected with TNBC [46].

In 2003, Hardy et al. found that saturated free fatty acids (FFA) decreased cell proliferation and induced apoptosis [47]. The saturated FFA palmitate decreased mitochondrial membrane potential, increased cytochrome c release, and increased caspase-3 activity. When treated with the acyl-CoA synthetase inhibitor triacsin C, the pro-apoptotic effect of palmitate was reduced, indicating that a certain level of metabolism of palmitate is required to induce apoptosis. The study also found that administration of palmitate led to a subsequent depletion of cardiolipin within the mitochondrial membrane. Thus, leading to decreased cytochrome c retention [47].

Guo et al., 2015, investigated the use of selenium yeast on TNBC and ER+ breast cancers [48]. Selenium yeast led to early apoptosis and disruptions in the mitochondrial membrane potential in MDA-MB-231 cells. However, it did not affect non-malignant cells. Selenium metabolism may lead to increases in reactive oxygen species, which may in turn cause the observed decrease in mitochondrial membrane potential [48].

## 4. Discussion

Targeting altered metabolism in cancer cells provides an approach that could lead to individualized treatment options, given that cancer cells display many unique metabolic characteristics as compared with healthy cells [49]. Therapies that target the metabolic features of breast cancer cells while having little or no effect on nonmalignant cells could lead to therapeutic options with high patient tolerability. A study by Gupta in 2014 showed that in a cross-sectional survey of 160 women with HR+ and HER2- breast cancer, those using hormone therapy reported greater health-related quality of life and treatment satisfaction than women receiving chemotherapy, indicating the benefit of targeted breast cancer treatment over chemotherapy [50]. While research is still in the early stages, targeting cancer cell metabolism could give a more targeted treatment option for TNBC.

**Figure 1:**
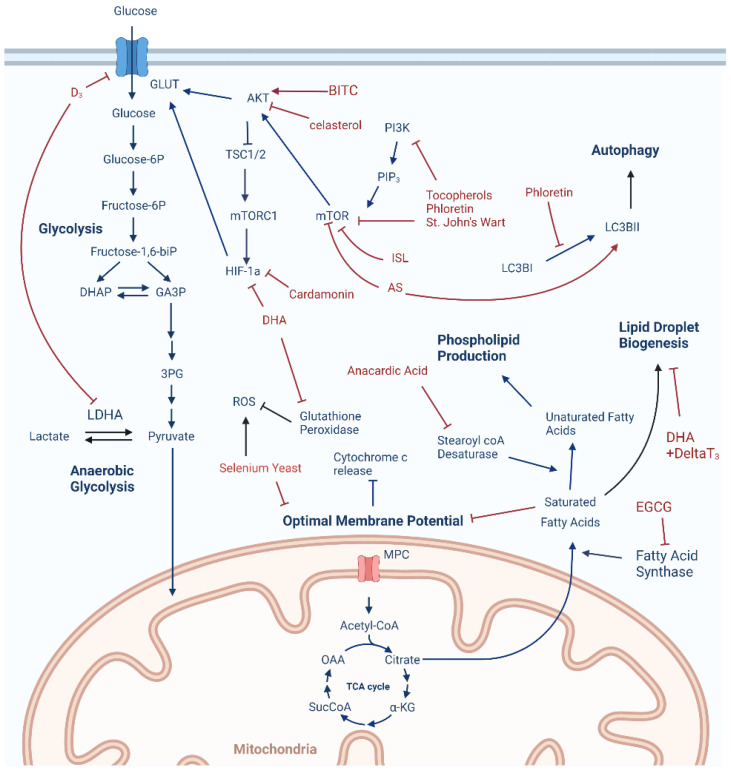
Metabolic targets of nutrients and nutraceuticals in TNBC cells. Akt, protein kinase B; AS, Antrodia Salmonea, BITC, benzyl isothiocyanate; D_3_, vitamin D_3_ (calcitriol); Delta T_3_, vitamin E delta-tocotrienol; DHA, docosahexaenoic acid; DHAP, dihydroxyacetone phosphate; EGCG, epigallocatechin gallate; GA3P, glyceraldehyde-3-phosphate; GLUT, glucose transporter; HIF-1a, hypoxia inducible factor 1 alpha; ISL, isoliquiritigenin; LDHA, lactate dehydrogenase A; MPC, mitochondrial pyruvate carrier; mTOR, mammalian target of rapamycin; mTORC1, mammalian target of rapamycin complex 1; OAA, oxaloacetate; PI3K, phosphoinositide 3-kinase; PIP_3_, phosphatidylinositol (3,4,5)-trisphosphate; ROS, reactive oxygen species; SucCoA, succinyl-coenzyme A; TSC1/II, Tuberous sclerosis I/II; 3PG, 3-phosphoglycerate; α-KG, alpha ketoglutarate.

Our goal with this review was to understand what is known about how nutrients or nutraceuticals target TNBC cell metabolism to exert anticancer effects. Figure 1 and Table 1 summarizes the key findings from each of the studies included in this review. It should be noted that although there were systematic elements for our searching and review methods, this review is primarily a narrative review that focuses on what is currently in the literature about how nutrients/nutraceuticals specifically target metabolism or metabolic processes in TNBC. Any potential biases of these studies, including outcome biases, should be considered when assessing the current state of this field.

A key finding from this review is that this information currently comes from pre-clinical models, with most results coming from in vitro studies. This indicates that these effects have not been validated in humans and therefore, this information should not be used in its current state to guide clinical treatment of TNBC. Instead, this information can be used to guide more in-depth studies for these compounds, guide studies that investigate other nutrient/nutraceutical anticancer mechanisms, or guide the development of more potent/specific agents that mimic the actions of these compounds. Much more work needs to be done with these compounds such as establishing safety in humans, effects at different dose ranges, interaction effects with established treatment regimens, and differential effects in subpopulations. The importance of considering the potentially harmful effects of nutritional supplements on cancer therapy has been discussed at length elsewhere [51]. In particular, the use of antioxidants during chemotherapy has also been noted to have detrimental effects [52]. These observations should be taken into account when considering the potential of these compounds as many have antioxidant properties. Along the same lines, it is well known that cancer cells have higher levels of ROS and antioxidant defenses as compared with normal cells [53]. Nutrients/nutraceuticals, particularly those that have proven ROS-modulating activity, may be able to leverage this property of cancer cells to exert cytotoxic effects. However, targeting of these compounds to tumor sites may prove difficult, and their effects on healthy cells should be considered in future research.

Overall, several different classes of nutrients have been found to disrupt triple-negative breast cancer metabolism/metabolic signaling and induce TNBC cell death. While some studies demonstrated evidence that individual nutrients induced dysregulation, other studies provided evidence that nutraceuticals used in conjunction with chemotherapeutics resulted in dysregulation. In the 2019 study by Roy et al., administration of BITC led to AKT phosphorylation and sugar dependency, and pharmacological inhibition of AKT led to greater decreases in cell viability [24]. Additionally, the 2021 study by Chen et al. found that administration of phloretin, a dihydrochalcone found in apples, caused increased sensitivity to doxorubicin in mice transfected with the MDA-MB-231 cell line [40]. Lastly, the study by Vibet et al. in 2011 indicated that DHA supplementation sensitized TNBC cells to anthracycline treatment, increased levels of reactive oxygen species, and downregulated the activity of glutathione peroxidase [34]. These studies give further evidence that components of the diet or a prescribed nutrient cocktail may make current chemotherapeutics more effective, although more research in this area is needed.

DHA in particular has been investigated in multiple studies regarding its anticancer activity through targeting cancer cell metabolism. Mouradian et al. in 2015 found that administration of DHA in MDA-MB-231 cells led to decreases in hypoxia-inducible factor, lactate dehydrogenase activity, and glucose uptake [25]. Pizato et al. found that DHA administration alone led to increases in lipid droplet biogenesis which contributes to TNBC malignancy, however, co-treatment with Delta T-3 led to decreases in lipid biogenesis and increases in lipophagy [33]. Lastly, Vibet et al. found that DHA administration sensitizes TNBC cells to anthracyclines, leading to increased ROS levels and decreased glutathione peroxidase activity [34]. These three studies give some evidence that DHA may lead to TNBC cell death and metabolic dysregulation through multiple pathways.

Another insight is the unclear role of saturated fatty acids in TNBC growth and development. Xiao et al. found that leucine deprivation inhibited FAS and led to decreased viability of TNBC cells [30]. However, supplementation with palmitate reversed this inhibition. Crous-Maso et al. also found that inhibition of FAS by EGCG led to decreased cellular viability, and reversal of this effect by reintroducing palmitate [31]. Hardy et al. however, found that saturated FFA such as palmitate led to increases in apoptosis and unsaturated fatty acids allowed for cellular proliferation and growth [47]. More research is needed to investigate the mechanisms by which saturated fatty acids such as palmitate act on TNBC cells and the therapeutic potential of these compounds.

To further understand the impact of diets, supplementation, natural products, and nutraceuticals as treatments or co-treatments for triple-negative breast cancers, further human subject research, as well as studies using in vitro and in vivo animal models are needed. Because it is known that essential nutrients that are obtained through diet are needed for the proper transcription and regulation of DNA, protein folding, and providing the co-factors needed for host metabolism, these nutrients should be investigated in combination, as well as individually, to target multiple metabolic perturbations that result after cancer initiation and proliferation. Indeed, it is well-recognized that effective treatment of cancer requires a “multi-hit” strategy, and most cancers are treated clinically with the administration of multiple agents. This is largely due to the adaptability of cancer cells, allowing the development of treatment resistance by upregulating/downregulating certain pathways or molecular targets to reduce the efficacy of a particular therapeutic agent [54]. This observation holds true for therapeutics targeting cancer cell metabolism as well.

Because of this, a combination—or cocktail—of nutrients/nutraceuticals is likely to be the most successful strategy in targeting cancer. This strategy has been proposed elsewhere for other indications [55]. As discussed in this review, many of these agents are known to target key metabolic processes that cancer cells rely on. By targeting multiple metabolic pathways simultaneously, cancer cells would be less likely to shunt metabolism to alternate pathways to survive this metabolic stress, which would in turn lead to a greater amount of cancer cell death. Importantly, many of these agents described above were shown to have favorable or nontoxic effects to nonmalignant cells, indicating the potential for a larger therapeutic window as compared with conventional chemotherapeutics.

The potential of combining nutrients/nutraceuticals with existing drugs should also be considered. Some classes of compounds, such as polyphenols and omega-3 polyunsaturated fatty acids (PUFAs), have been shown to increase the efficacy of many anticancer drug classes such as anthracyclines, platinum drugs, and nucleoside analogs—classes commonly used to treat TNBC [56,57]. Some of these compounds, particularly omega-3 PUFAs (including DHA), have also been shown to reduce the side effects associated with chemotherapeutics including cancer-associated cachexia [58]. This indicates that not only can nutrients/nutraceuticals increase cancer drug efficacy, they can also diminish the dose-limiting side effects of these compounds, allowing for the de-escalation of chemotherapy, or the use of more effective doses. The combination of nutrients with cancer drugs has already been shown to be successful clinically, with the combination of folinic acid with fluorouracil and oxaliplatin for the treatment of colon cancer, also known as the FOLFOX regimen [59]. Additional studies are also needed to see if a synergistic effect can be achieved by combining the agents reviewed in this article with known therapeutics. For example, increased glucose dependence caused by BITC treatment could be combined with a ketogenic diet to potentially increase its anticancer effect. Another example could be the combination of celasterol with other mTOR inhibitors (e.g., everolimus) to potentiate its anticancer effect. These types of studies will be highly important to better understand how these—or similar—compounds interact positively (or negatively) with existing therapies. Given that untargeted metabolomics methods exist for TNBC cell experiments [60], more comprehensive investigations at how nutrients/nutraceuticals, alone or in combination with existing therapeutics, affect TNBC cells can be performed.

In conclusion, TNBC displays a number of metabolic adaptations that allow for the initiation and progression of this disease. Nutrients and nutraceuticals have been demonstrated to have anticancer effects towards TNBC and they conduct these effects through targeting cancer cell metabolism. In some cases, the effects of these compounds show selective toxicity towards cancer cells, indicating the potential for less severe side effects as compared with other anticancer agents. Combining nutrients/nutraceuticals into cocktails—along with anticancer drugs—would provide a strategy to target multiple metabolic adaptations simultaneously, potentially elevating efficacy far beyond each agent alone. Furthermore, many of these compounds may aid in reducing side effects, making treatments more tolerable. More research is needed to better understand how these agents interact with established cancer therapies and any other relevant side effects. The current state of research of these agents is still in the early stages, and more work needs to be done in more translatable model systems to better understand the potential of these compounds alone and in conjunction with other therapies.

## Figures and Tables

**Table 1:** Summary of reviewed studies.

Reference	Author (Year)	Intervention	Dietary Counterpart	Model	Pathway	Mechanism
22	Roy (2019)	Benzyl Isothiocyanate	Mustard Family	MDA-MB-231 cells + Mice	Glycolysis	↑: GLUT-1 localization, AKT activity
23	Shrivastava (2015)	Celasterol	Tripterygium wilfordii and Tripterygium regelii	MDA-MB-231 cells	Glycolysis	↓: AKT activity
						↑: apoptosis
24	Jin (2019)	Cardamonin	Alpiniae katsumadai	MDA-MB-231 cells + Mice	Glycolysis	↓: HIF-1a expression, glucose uptake, lactic acid production;
						↑: ROS production
25	Mouradian (2014)	DHA	DHA	BT-474 and MDA-MB-231 cells	Glycolysis	↓: HIF-1a expression, LDHA, lactic acid, glucose uptake
26	Santos (2018)	Calcitriol	Vitamin D3	MDA-MB-231 and MCF-7 cells	Glycolysis	↓: GLUT-1 expression, LDHA expression, HKII expression, lactate concentration
30	Xiao (2016)	Leucine	Amino Acid/Protein	MDA-MB-231 and MCF-7 cells + Mice	Fatty Acid Metabolism	↓: FAS expression, Sterol Response Element Protein CII, Palmitate
31	Crous-Maso (2018)	EGCG	Green Tea, fruits	MDA-MB-231 cells	Fatty Acid Metabolism	↓: FAS expression, palmitate
34	Schultz (2018)	Anacardic Acid	Cashews	MCF-7 and MDA-MB-231 cells	Fatty Acid Metabolism	↓: Stearoyl coA desaturase expression
37	Pizato (2019)	DHA + Vitamin E Delta-T3	DHA, Vitamin E	MDA-MB-231 cells	Fatty Acid Metabolism	↑: Lipid droplet lipophagy
						↓: Lipid droplet formation
40	Vibet (2011)	DHA + Anthracyclines	DHA	MDA-MB-231 and MCF-7 cells + Mice	Oxidative Stress	↑: GSH accumulation, ROS
						↓: GPx activity
41	Tran (2015)	Tocotrienols, Tocopherols	Vitamin E	MCF-7 and MDA-MB-231 cells	Autophagy/Apoptosis	↑: autophagy, apoptosis
						↓: mTOR and PI3K activity, cell proliferation
42	You (2020)	St. John’s Wort	Hypericum perforatum	MDA-MB-231 cells + Mice	Autophagy/Apoptosis	↑: pro-death autophagy
						↓: mTOR and PI3K phosphorylation
43	Lin (2020)	Isoliquiritigenin	Licorice	MDA-MB-231 cells + Mice	Cell Cycle Arrest	↑: Bax protein expression
						↓: mTOR phosphorylation, Cyclin D1 expression, Bcl-1 protein
44	Chang (2017)	Antrodia Salmonea	Fungus	MDA-MB-231 cells + Mice	Cell Cycle Arrest	↑: LC3B-II, caspase-3
						↓: Cyclin B1, cyclin A, cyclin E, CDC2, COX protein expression
45	Chang (2017)	Antrodia Salmonea	Fungus	MDA-MB-231 cells + Mice	Oxidative Stress	↑: LC3-II, AVOs formation, apoptosis
						↓: mTOR phosphorylation
46	Chen (2021)	Phloretin	Apples	MDA-MB-231 cells	Autophagy/Apoptosis	↓: LC3-I to LC3-II conversion, ULK1 expression; ↑: mTOR and AMPK phosphorylation, sensitivity to doxorubicin
47	Hardy (2003)	Saturated Free Fatty Acids	Fatty Acids	MDA-MB-231 cells	Autophagy/Apoptosis	↑: apoptosis, cytochrome c relase, caspase-3 activity
						↓: cell proliferation, mitochondrial membrane potential
48	Guo (2015)	Selenium Yeast	Selenium Yeast	MDA-MB-231 and MCF-7 cells	Autophagy/Apoptosis	↑: apoptosis
						↓: disruption of mitochondrial membrane potential
